# Assessment and comparison of the pathogenicity of Sheeppox Virus strains isolated in Morocco

**Published:** 2017-12

**Authors:** Saida Hajjou, Khadija Khataby, Souad Amghar, Mustapha El Fahime, Mehdi El Harrak, Malika Fakiri, Chafiqa Loutfi

**Affiliations:** 1 Laboratory of Agro-Food and Health/Genetics & Biotechnologies Team, Faculty of Sciences and Technology, University Hassan I, Settat, Morocco; 2 Laboratory of Virology, Microbiology, Quality and Biotechnologies/Ecotoxicology & Biodiversity, Faculty of Sciences and Techniques, University Hassan II of Casablanca, Morocco; 3 Society Biopharma, Km 2, Route de Casa, B.P. 4569, Rabat, Morocco; 4 Department of Biological Analysis/PGF/UATRS/CNRST, BP 8027 NU, 10102, Rabat, Morocco

**Keywords:** Sheep pox, Sheep poxvirus, Morocco, Pathogenicity

## Abstract

**Background and Objectives:**

Sheeppox virus causes systemic disease in sheep that is often associated with high morbidity and mortality. Protection against sheep pox is mainly based on medical prophylaxis, vaccination being the only way. In Morocco, and up to now, there is no available information about local challenge strain to use for controlling the efficiency of vaccines produced against sheep pox. Hence, the objective of the present study was to evaluate and compare the pathogenicity of seven Sheeppox virus (SPVs) isolates from 1993–1995 in Morocco.

**Materials and Methods:**

These seven SPV isolates have undergone various tests to evaluate their pathogenicity: Passages and titration on cell culture, Experimental inoculation on sheep, Virus-neutralization, *In vivo* titration and viral re-isolation by real-time PCR assay.

**Results:**

All infected lambs showed severe clinical signs, while most of them have been reproduced on 5 dpi and persisted until 21 dpi. The lambs infected by Oj1P4, Oj2P4 and BerP5 appeared lethargic, reluctant to move compared to those infected by other isolates. The results also revealed that all isolates were able to induce serological response. Virus isolation from infected organs and blood and amplification of the viral DNA by real-time PCR proved the presence of the virus in tissues and blood of infected lambs. These Moroccan SPVs demonstrated that the three isolates Oj1P4, Oj2P4 and BerP5 have a high pathogenicity; especially the BerP5 isolate which has an important infectious titer.

**Conclusion:**

These results demonstrate that the Berkane isolate is the most pathogenic of the tested isolates and it can be an excellent challenge strain for the control of the efficiency of vaccines against sheep pox produced in Morocco.

## INTRODUCTION

Sheeppox (SP) is an infectious and highly viral contagious disease of small ruminants that is endemic in the near East and Central Asia, India, China and Central and Northern Africa (1, 2). The disease is responsible for serious economic losses to developing country such as Morocco, affecting Sheep of all ages and categorized as a notifiable disease by the World Organization for Animal Health (OIE).

The causative viral agent of SP is a member of Capripoxviruses (CaPVs) genus, belongs to the Poxviridae family. The genus Capripoxvirus comprises three members namely, sheeppox virus (SPV), goatpox virus (GPV) and lumpy skin disease virus (LSDV) affecting sheep, goats and cattle, respectively (3). They are closely related, showing 96% and 99% identities between the members of the same genus (4).

SPV has a wide geographical distribution and it was found in regions of Africa, Middle East and Asia, where it has a major economic impact on small ruminants production due to the high morbidity and mortality associated with disease in susceptible sheep (5). It causes significant economic losses via reduced milk yield, decreased weight gain, increased abortion rates in pregnant animals, damage to wool and hides (can affect as much as 50% of the skin surface), increased susceptibility to pneumonia and other secondary infections. Sheep of all ages can be infected with the sheeppox virus (SPV), and infected sheep show fever, pyrexia, rhinitis, conjunctivitis, excessive salivation and generalized pock lesions in the skin (3, 6).

Besides, the fact that CaPV infections cannot be distinguished clinically or serologically emphasizes the need to establish more reliable tests for virus identification such as one based on a molecular method like PCR, cloning and sequencing which are more reliable, sensitive and specific techniques (7, 8).

In Morocco, for many decades, sheep pox has been identified in enzootic form (9). Since the discovery of the disease in Morocco, regular control programs undertaken by the National Veterinary Services of the Department of Agriculture have been carried out in order to control the disease (9). These programs aimed to eradicate SPV by using a local live vaccine (10). This vaccine confers a long-lasting immunity; gives an immunity that can reach more than 24 months in vaccinated animals.

Given the significant economic losses of sheeppox worldwide and in Morocco, unfortunately up to now, there is no available information about local virus challenge. Hence, the objective of the present study was to evaluate and compare for the first time the pathogenicity of seven Sheeppox virus strains isolated during 1993–1995 in Morocco. The clinical signs, gross lesions were evaluated. Serological response by the detection of SPV antibodies of the affected lambs was also checked. Isolation of the virus from the affected organs and real time PCR test was used to detect virus in several tissues of infected sheep.

## MATERIALS AND METHODS

### Virus isolates.

It’s about a seven Moroccan virulent isolates of SPV (Table 1), which have been isolated from sheep suffering from the specific clinical signs of SP and which had been previously isolated in Biopharma laboratories during 1993–1995 severe epizootics of SPV in different geographic regions of Morocco.

**Table 1.** Presentation of isolates: designation, origin and outbreak year.

**Designation**	**Origin**	**Outbreak Year**
Oj1P2	Oujda	93/10/20
Oj2P3	Oujda	93/11/15
BerP3	Berkane	93/11/15
Tac2P2	Taounate	94/06/03
Hz2P2	Haouz	94/11/07
ErrP1	Errachidia	94/11/07
Azi2P1	Azilal	95/02/21

Initially, biopsy specimens were homogenized in PBS (10% w/v) containing 1% AB solution and 200 μl of biopsy suspensions was used to infect mono-layer cells grown in 25 cm^2^ flasks in duplicate with negative controls. Cells were examined daily for cytopathic effect (CPE) for 7–12 days after infection (dpi) (11). The contents of CPE-negative flasks were collected and freeze-thawed and were again used to infect fresh cells, which were examined daily for another 7–12 dpi for CPE. Samples had no CPE after the 6th passage; were considered virus isolation negative. The passage number of each isolate is reported in the designation (Table 1). The different isolates were frozen at −80°C and maintained in the laboratory as a cell culture forms.

### Selection and identification of pathogenic field isolates.

Prior to inoculation of experimental animals, all stored isolates were screened by qPCR to detect SPV genomes to confirm the virus identity and then revived in diploid cells to confirm the presence of infectious virus after long term storage.

A one step Real time PCR was carried out using Invitrogen kit (SuperScript® III Platinum, Life Technologies, USA). The DNA amplification was performed using universal pair of primers a) downstream primer, CAPV-074F1, targeting gene encoding to envelop protein P32 (5′AAA ACG GTA TAT GGA ATA GAG TTG GAA-3′); and b) upstream primer, CAPV-074R1, 5′-AAA TGA AAC CAA TGG ATG GGA TA-3′, and the Taqman probe: 5′-FAM-TGG CTC ATA GAT TTC CT-MGBNFQ-3′ (12). The amplifications reactions (PCRs) were performed on a Smart Cycler. The following mix of each reaction was contained: 12.5 μl 2× PCR buffer mix, 0.5 μl MgSO_4_ (50 mM), 0.5 μl Rox (25 mM), 4.75 μl nuclease free water, 0.5 μl primers to a final concentration of 10 μM, 0.25 μl probe to a final concentration of 10 μM and 5 μl DNA template. The reaction was carried out in Step One TM Plus real-time PCR system (Smart cycler Cepheid, USA) at 94°C for 5 min, 94°C for 10 min, and 40 cycles of 55°C for 15s and 72°C for 45s. All reactions amplifications were recorded, analyzed, and the threshold cycle (Ct) determined with the Step One software (Smart Cycler).

### Virus titration.

For virus titration 6-fold dilutions (10^−1^ to 10^−6^) were inoculated into the sheep heart cell carcinoma (ICO1 cell lines), and incubation during 7–10 days at temperature of 37°C +/− 2°C and relative humidity of 75% +/− 10%. The titer is calculated and estimated as the reciprocal of the highest dilution of the virus giving 50% CPE according to the Spearman Karber formula (13, 14).

### Experimental infections.

In order to evaluate and compare the pathogenicity of Moroccan viral isolates, an experimental infection was performed in lambs; serologically negative (SN). For this purpose five local breed Lambs (2–4 months old) were housed in containment building with negative-pressure and water and feed were provided at *libidum*. The infection trials were approved by the local ethics committee and were carried out following strict animal welfare guidelines of Biopharma Society.

Using a syringe, lambs were inoculated intradermally with SPV into shaved area on the left flank over the last rib with 20 mL of tissue culture; containing 10^5^ TCID50/ml of either OJ1P4, Oj2P4, BerP5, Azi2P3 or Tac2P3 isolates, and one control lamb was inoculated with phosphate-buffered saline (1XPBS) (13).

The specific clinical signs of SP were recorded daily during 21 days post-infection (dpi). Specific clinical signs of SP observed included nasal discharge, depression, pyrexia, rhinitis, conjunctivitis, and excessive salivation. Each day (dpi), rectal temperatures were also recorded and animals were examined for lesions development at the inoculation site.

### Serological response.

Virus-neutralization test (VNT) was carried out on serum samples collected from experimentally infected lambs for antibodies detection at 0, 7 and 14 dpi. The test was performed out with constant virus (100 TCID 50/ml) and variable serum (dilutions of 2 in 2) using 96-well flat-bottomed tissue culture grade microtiter plates seeded with monolayer cells. After 10 days of incubation at 37°C, the neutralizing titer (index) is calculated by the method of Reed and Muench (15). Moreover, serology on SN lambs from the same rearing was also performed to ensure the negative status anti-SPV lamb’s antibodies used.

### Viral re-isolation.

At 0, 3, 6 and 9 dpi; whole blood was collected from each lamb into EDTA-tubes to detect SPV genomes by qPCR assay as described previously. Lambs were euthanized when they were moribund. Tissue samples (lymph tissues and skin lesion samples collected at necropsy) were collected and immediately frozen at −80°C until use for virus isolation, *in vivo* titration or DNA extraction.

### *In vivo* virus titration.

In order to determine the recreational viral dose, biopsy materials were collected from each necropsied animals infected with Oj1P4, Oj2P4 and BerP5 isolates. Samples were ground and homogenized in PBS supplemented with 5% antibiotics, as described above. Three additional SN lambs, were used in this experiment; one lamb for each virus. For *in vivo* titration, 7 fold serial dilutions ranging from 10^−1^ to 10^−7^ were used in this study. Using a syringe, lambs were intradermally administrated with 0.1 mL of inoculums of virus isolate into five sites for each dilution (13). Each injection site was examined daily for evidence of macula development. The viral titer was determined at 7 dpi and was estimated as the reciprocal -index- of the highest dilution of the stock virus giving 50% reactive dose [RD50] after Spearman Karber method (13).

## RESULTS

### Selection and identification of virulent field isolates.

The identification of SPV field isolates was assessed initially using qPCR to detect specific SPV and DNA extracted from pure cultures confirmed the presence of SPV genomes in all samples (Table 2).

**Table 2.** Results (threshold cycle value) of real-time PCR obtained from pure culture of SPVs on monolayer cells.

**Isolates**	**Oj1P2**	**Oj2P3**	**BerP3**	**Tac2P2**	**Hz2P2**	**ErrP1**	**AziP1**
Ct value	21.85	22.39	22.69	24	30.35	24.44	28.58

### Virus titration.

The isolated strains Oj1P2, Oj2P3, AziP3, Tac2P2 and BerP3 have been subsequently passaged in sheep heart cell carcinoma (ICO1 cell lines) prior to *in vitro* titration. These subsequent passages showed a characteristic and reproducible CPE which initiated by 2–3 day cell post-infection and progressed to 80% by days 6–8. These isolates were therefore selected for quantification of infectious viral particles. BerkP5 demonstrated the highest infectious titer of 7.32 log TCID50/ml at its 5th passage. Strains Oj1P4, Oj2P4 and AziP3 had the same titer of 6.8 DICT50/ml, while Tac2P3 had the lowest titer of 5.8 DICT50/ml. ErrP1 and Hz2P2 isolates were discarded because they did not manifest any CPE. According to the obtained results, Oj1P4, Oj2P4, AziP3, Tac2P5 and BerP5 were therefore selected to study SPV pathogenesis in lambs.

### Clinical signs and gross pathological findings: Rectal temperature.

**Fig. 1.**
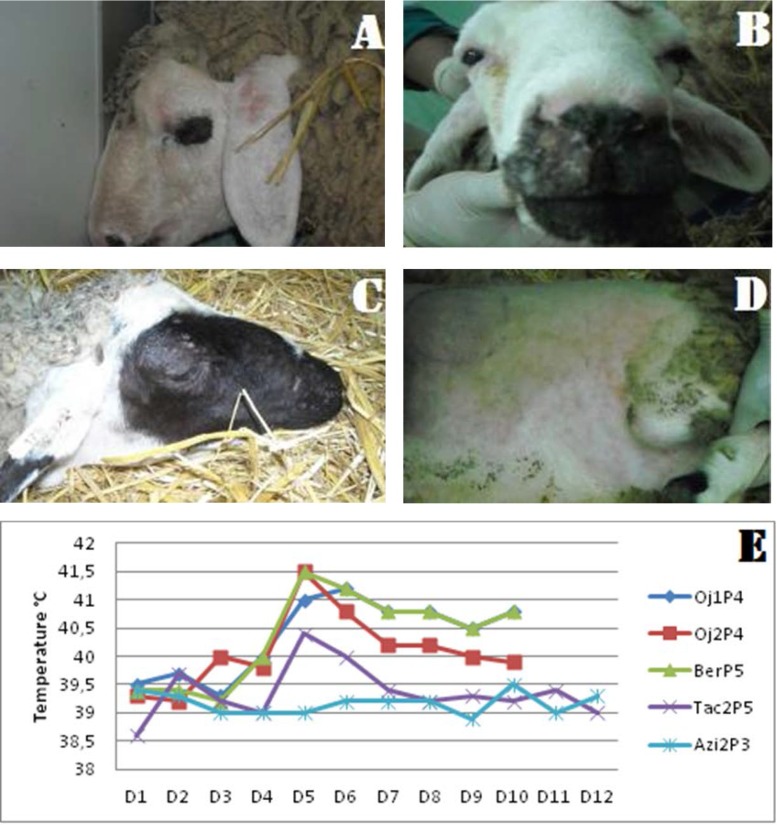
Clinical sings and rectal temperatures obtained from infected animals.(A) Papules in the ears and facial edema, (B) Nasal secretions, (C) Crusts on the head of infected animal, (D) Generalization of the disease, (E) Daily rectal temperatures of sheep infected with the 5 strains.

During the entire period of the experiment, the lamb infected with AziP3 isolate, showed stable rectal temperature (rt) (39°C) (Fig. 1-E). At 4 dpi, all other lambs were developed moderate fever [rt>41°C]. As shown in the Fig. 1, rectal temperature increased up to 41°C for animals inoculated with Oj1P4, Oj2P4, Tac2P5 and BerP5 isolates, which is coincided with the development of erythematous macules. By 10–12 dpi, normal values of rectal temperature were recorded in sheep infected with Oj2P4 and Tac2P5, while fever persisted for up to 10 dpi in animals infected with Oj1P4 and BerP5.

### Clinical signs.

Clinical monitoring of infected lambs reveals at first, apparent pathognomonic sings beginning at 5 dpi. The most prominent clinical signs were skin macules that progressed into papules throughout animal’s body and crusts on the head (Fig. 1-a, b, c and d).

In the most severely affected animals, papules (Fig. 1) were distributed over more than 50% of the skin surface and were most noticeable in less well-haired or wooled regions including the axillae, groin, perineum, ventral surface of the tail, muzzle and ears, with that exception for lambs infected by the three viral isolates (Oj1P4, Oj2P4 and BerP5) showing more severe signs (100%) along the study period.

Other developed clinical signs included nasal discharge, rhinitis, conjunctivitis and excessive salivation. Besides, superficial lymph node enlargement and petechiation were also observed at this time and were particularly prominent in prescapular nodes. These clinical symptoms persisted in all groups until 21 dpi.

At autopsy, all infected lambs that were sacrificed at 21 dpi showed typical pox lesions on buccal mucosal surfaces (tongue and lips). During the experiment, no clinical signs or gross lesions were observed in the non-infected control lamb.

### Skin lesions development.

**Fig. 2.**
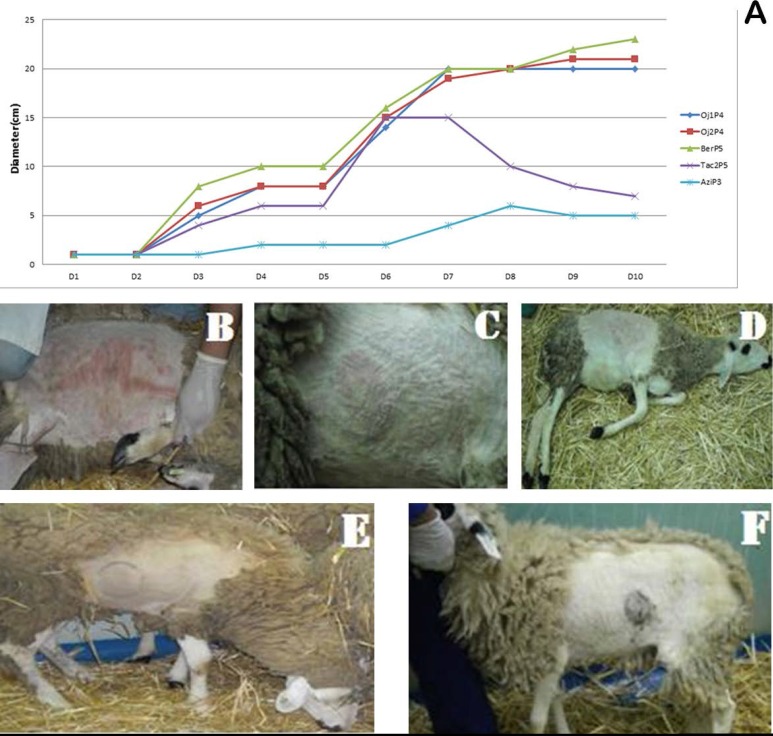
(A) Diameter of local reaction of inoculated animals, (B) BerP5, (C) Oj1P4, (D) Oj2P4, (E) TaC2P5, (F) Azi2P3.

Within 3 days following inoculation, primary large local reactions were developed in 8–9 cm diameter swelling at the injection site for different isolates (Fig. 2). However, the progression to macules was different depending on the isolates. Indeed, macules were seen by 8–9 dpi with significant swellings of up to 20–22 cm in diameter for animals inoculated with Oj1P4, Oj2P4 and BerP5 isolates, and macules progressed to papules within 1 to 2 dpi until last days of the experiment.

Moreover, the appearance of multiple secondary papules over the body was observed in the most severely affected animal which was inoculated with BerP5 isolate. In contrast, Ta2P5 and Azi2P3 isolates produced a soft generalized reaction, the skin lesions faded rapidly and were no longer visible at 7 dpi on animals infected with these isolates.

### Viral re-isolation.

Results related to SPV re-isolation was checked in all blood and tissue samples collected at 0, 3, 6 and 9 dpi of all infected lambs. The virus was found in the lymph nodes and skin of animals infected with Oj1P4, Oj2P4 and BerP5 viruses, showing significant Ct values ranged from 17.18 to 35.54 (Table 3).

**Table 3.** Results (threshold cycle value) of real-time PCR obtained from Blood, Skin lesions and Lymph nodes of infected animals.

	**Samples**	**qPCR results (Ct)**		**Samples**	**qPCR results (Ct)**
Blood	BerP5/D0	37.47	Blood	Azi2P3/D0	39.59
	BerP5/D3	39.70		Azi2P3/D3	37.86
	BerP5/D6	37.53		Azi2P3/D6	38.61
	BerP5/D9	45		Azi2P3/D9	38.68
	
	Oj1P4/D0	39.65		Tac2P5/D3	36.89
	Oj1P4/D3	43.03		Tac2P5/D6	37.02
	Oj1P4/D6	36.70		Tac2P5/D9	35.93

	Oj1P4/D9	36.45	Skin lesions	BerP5	18.15

	Oj2P4/D0	38.29		Oj2P4	17.25

	Oj2P4/D3	45	lymph nodes	BerP5	17.18
	Oj2P4/D6	37.80		Oj1P4	35.54
	Oj2P4/D9	37.58		Oj2P4	23.45

Small viral loads were detected by qPCR in collected blood samples. All samples became qPCR-positive prior to the appearance of clinical signs of disease, as defined by fever (Table 3). However, there was no significant difference in Ct value obtained between different samples, especially for those collected from animals infected with Oj1P4 and BerP5 viruses. These results were in accordance to the gross lesions such as hyperthermia during viremia (5–10 dpi).

The virus was isolated from all the sampled tissues with a high viral load in the lymph and skin. No SP virus was isolated from the control group. Real time PCR results obtained from the tissues of infected lambs was correlating in the positiveness with those obtains from blood samples.

### Serological response.

Blood samples, collected from each lamb were analyzed by VNT for anti-SPV antibody titers. Serological responses induced by the SPV isolates as determined by VNT are presented in Table 4. The VNT performed at 7 and 14 dpi, showed the presence of antibodies, with a titer as high as 0.9 at 7 dpi. This result confirms that infected lambs seroconverted against SP, after manifestation of specific clinical signs; especially a high representative titer was noted for BerP5 isolate (Table 4).

**Table 4.** Titers of neutralizing anti-bodies acquired from the 5 inoculated sheep.

	**Oj1P4**	**Oj2P4**	**BerkP5**	**Ta2P5**	**AziP3**
0 Dpi	--	--	--	--	--
7^th^ Dpi	--	--	0.9	0.7	<0.66
14^th^ Dpi	1.02	1.26	1.38	0.9	--

### *In vivo* titration.

**Fig. 3.**
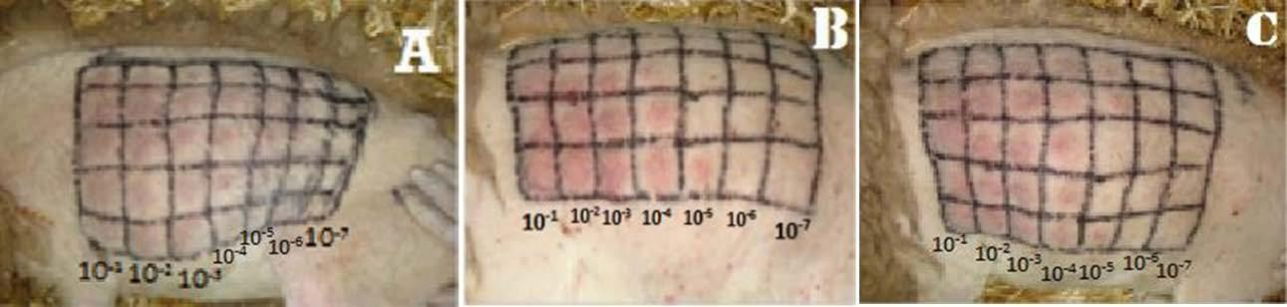
Results of titration *in vivo* by the viral strains: (A) BerP5, (B) Oj1P4, (C) Oj2P4

Virus titration of necropsied animals infected with Oj1P4, Oj2P4 and BerP5 isolates used in this study, was performed on three lambs. At 3 dpi, a local reaction produced in the injection site was observed (Fig. 3). At 9 dpi, the generalized reaction for all isolates was observed and occurred by the appearance of cutaneous nodules. According to the Spearman Karber formula, infectious titer for each virus homogenate is given in Table 6 and expressed as log RD50/ml. The results obtained by BerP5, Oj2P4 and Oj1P4 isolates demonstrate the highest titer of 6.7, 6.1 and 5.7 log RD50/ml, respectively.

## DISCUSSION

In the present work, the pathogenesis of seven Moroccan SPV isolates was investigated for the first time. No studies on pathogenicity of SP virus challenge were reported in neighboring Maghreb or other African countries. Previous investigations in Morocco focused only on molecular characterization of nodular form of SP (isolation and identification of virus responsible for the new clinical form of disease) (16), but no information was available about the pathogenicity and the tissue tropism of this virus. To do that, experimental infection in serologically negative lambs was used to evaluate pathogenicity and tissue tropism of seven Moroccan SPV isolates (coded: BerP5, Oj1P4, Oj2P4, Azi2P3, Tac2P5).

Lamb diploid cells were shown very sensitive to infections with isolates originated from Eastern parts of Morocco (Berkane and Oujda) where several outbreaks regularly occur despite vaccination. Indeed, after viral inoculation, infected cells developed a characteristic CPE from the second day (17). This result corroborates the results obtained by PCR assay. A second passage was required to reproduce the CPE for different isolates. Isolates collected from El Haouz and Errachidia, the central areas on Morocco did not show any CPE until the 6th day during both passages. These isolates did not retain their ability to infect monolayer cells and were not considered as potential virulent challenge viruses.

Clinical signs showed that all the five tested isolates of SPV, were capable to induce apparent pathogenic sings at 5 dpi. Infected lambs showed the various signs of depression, sneezing, difficulty in breathing, nasal discharge, skin lesions in axillae, groin, perineum, ventral surface of the tail, muzzle and ears. Macules progressed to papules throughout animal’s body and crusts on the head. Developed symptoms became severe at 5 to 15 dpi, and persisted in some lambs until 21 dpi. The lambs infected by Oj1P4, Oj2P4 and BerP5 appeared lethargic, reluctant to move compared to those infected by others isolates. The clinical signs, and gross lesions induced by this isolate are in accordance with the findings that have been described previously for other serotype of SPV (6, 12) and were in agreement with the clinical signs mentioned by others authors (10, 12, 13, 17–18).

Nevertheless, the differences in infectious titers, development and clinical appearance and immune response produced by different isolates suggest that the degree of pathogenicity and its severity (virulence) varies from one isolate to another. While Oujda and Berkane isolates were equally fever-inducing and immunogenic for sheep; the Berkane isolate was particularly virulent in sheep since it induced secondary lesions.

The highest concentrations of viral DNA were consistently detected at the inoculation site at swelling skin lesions for lambs inoculated with Oj1P4 and BerP5 isolates and, to a lesser extent, in peripheral blood for all tested lambs. This was in agreement with previously reported data (2, 16, 19–20). Viremia in SPV infected animals is transient and uniformly low according to Bowden et al. (2008) (12) and Balinsky et al. (2008) (21). The high level of neutralizing antibodies obtained by 10 dpi for Berkane isolate (i.e. 1.34) indicates virus multiplication in the organism rather than a mean of protection since in terms of sheep pox, immunity is mainly conferred through cellular response (13, 22). The Oujda and Berkane districts are regions where the disease had a significant economic impact and where animals perform regularly migratory movements.

Results of virus isolation confirm SPV distribution in tissues and blood samples examined at 0, 3, 6 and 9 dpi, with the five tested isolates. These findings were in accordance with those of other investigations on virus isolation (12, 16).

In this report, VNT test used to evaluate levels of the AB to SPV in all infected lambs at 0, 7, and 14 dpi. This result confirms that infected lambs were seroconverted against SP, after manifestation of specific clinical sings. Indeed, as described in OIE Manuel of diagnostic (2008), VNT could be reliable, repeatable, and sensitive for detecting SP antibodies (11).

On the other side, *in vivo* titration, using BerP5, Oj1P4 and Oj2P4 isolates showed the generalization of the infection, which characterized by cutaneous manifestations, expansion of the diameter of inoculation sites, and hyperthermia appearing on the 5^th^ dpi for the BerP5 isolate, and at the 6 and 7 dpi for Oj1P and Oj2P4, the same results were obtained by Achour et al. (2000) (13). The titers obtained are 5.7 DR50 / ml for Oj1P4, 6.1 DR50 / ml for the Oj2P4 and 6.7 DR50 / ml for BerP5 isolate.

Results of *in vitro* titration showed 5.8 TCID50 / ml for the TaCP2 isolate and 7.32 TCID50 / ml for the BerP5 isolate. According to Achour et al. (2000) (13), the *in vitro* titer was relatively low between 10^3^ and 10^5^ TCID 50, and between 10^−6.5^ and 10^−6.7^ for Sadri and Fallahi (2010) (23).

Results obtained with Moroccan SPV demonstrated that the three isolates Oj1P4, Oj2P4 and BerP5 have a high pathogenicity with strong relationship between them, especially the BerP5 isolate which has an important infectious titer.

In conclusion, current report justify that the Berkane isolate is the most pathogenic of the tested Moroccan SPV isolates, and it can be an excellent challenge virus for the control of the efficiency of vaccines against sheep pox produced in Morocco.
